# World Aquaculture: Environmental Impacts and Troubleshooting Alternatives

**DOI:** 10.1100/2012/389623

**Published:** 2012-04-29

**Authors:** Marcel Martinez-Porchas, Luis R. Martinez-Cordova

**Affiliations:** ^1^Departamento de Tecnología de Alimentos de Origen Animal, Centro de Investigación en Alimentación y Desarrollo, Km. 0.7 Carretera a La Victoria, Hermosillo, SON, Mexico; ^2^Departamento de Investigaciones Científicas y Tecnológicas de la Universidad de Sonora, Boulevard Luis Donaldo Colosio s/n, 83000 Hermosillo, SON, Mexico

## Abstract

Aquaculture has been considered as an option to cope with the world food demand. However, criticisms have arisen around aquaculture, most of them related to the destruction of ecosystems such as mangrove forest to construct aquaculture farms, as well as the environmental impacts of the effluents on the receiving ecosystems. The inherent benefits of aquaculture such as massive food production and economical profits have led the scientific community to seek for diverse strategies to minimize the negative impacts, rather than just prohibiting the activity. Aquaculture is a possible panacea, but at present is also responsible for diverse problems related with the environmental health; however the new strategies proposed during the last decade have proven that it is possible to achieve a sustainable aquaculture, but such strategies should be supported and proclaimed by the different federal environmental agencies from all countries. Additionally there is an urgent need to improve legislation and regulation for aquaculture. Only under such scenario, aquaculture will be a sustainable practice.

## 1. Introduction

**Figure 1:**
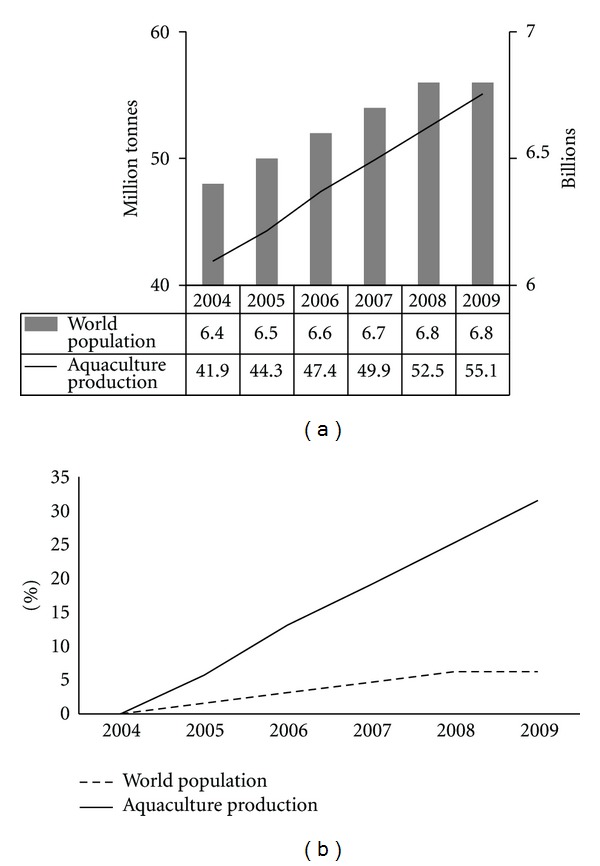
Growth behavior of world population and aquaculture production during the last six years. (a) illustrates the total world population by year (billions) and the total production of aquatic organisms by aquaculture (million tonnes). (b) compares the percentage of annual increase of world population and aquaculture production, considering year 2004 as the basepoint. Data obtained from FAO Report 2010: World Review of Fisheries and Aquaculture.

Aquaculture, the farming of aquatic organisms, has been the agroindustrial activity with the highest growth rate worldwide in the last four decades. From 1970 to 2008 the production of aquaculture organisms grew at a rate of 8.3% per year, compared to less than 2% of fisheries, and 2.9% of livestock [1]. The annual aquaculture production is at present over 60 million tons (including marine plants), with an approximate value of 85 billion dollars [2]. The last FAO report revealed that the world population increased by 6.3% from 2004 to 2009, whereas the production of aquatic organisms by aquaculture increased by 31.5% in the same period (Figure 1) [2].

 Despite the undeniable benefits of aquaculture such as the provision of good quality and accessible food for population and the generation of millions of jobs and billion dollars in budget for the developing countries, the activity is one of the most criticized worldwide, mainly because of the environmental impacts that have been and can be caused. Thus, the predominant and unavoidable question is: could aquaculture be a truly sustainable activity?

Understanding sustainability as “the ability to meet the needs of the present without compromising the ability of the future generations to meet their own needs” [3], many researchers, aquaculturists, and governmental instances have considered that a sustainable aquaculture is possible, but it depends on the way that the activity will be managed [4]. Additionally, other authors [5] have argued that “the sustainability of aquaculture not only requires neutral or benign effects on the environment, but also economic feasibility.”

The present paper is a review of the world aquaculture and its environmental impacts. It analyzes the situation of aquaculture production up to date and summarizes the main problems faced by the activity, as well as the strategies suggested, evaluated, and proven to contribute to achieve a sustainable activity.

**Aquaculture Is an Essential Activity for World's Welfare**

Aquaculture is considered as a double-edged sword, because it has not only tremendous benefits for the humanity, but also great repercussions to the environment. Considering the benefits, seafood produced by fisheries and aquaculture contributes with 15 to 20% of average animal protein consumption to 2.9 billion people worldwide [6] without considering the contribution of freshwater or brackish water species. The nutritional quality of aquatic products has a high standard and represents an important source of macro- and micronutrients for people from developing countries [7]. Additionally, aquaculture and fisheries are recognized as a source of employment; for instance, near to 43.5 million people were employed in 2006, and 520 million people relied on income from seafood production [6, 8].

Aquaculture products have also high trade potential as food commodities in the international market; fish and shellfish exports from developing countries have a greater value than the combination of important products such as coffee, tea, tobacco, meat, cocoa, rubber, and rice [6, 8]. In many cases, the incomes generated by aquaculture exceed those from other agricultural activities, due to the high price market that some products can achieve and due to the most effective bioenergetics of some aquatic species. However, despite all these benefits, aquaculture is actually not considered a sustainable activity in the perception of the scientific community and the average population.

## 2. Why Aquaculture Is Considered a Nonsustainable Activity?

With or without valid arguments, aquaculture has been accused to be the cause of many environmental, social, economic, and inclusively esthetic problems. Ecosystems are not always as fragile as could be considered, instead, they have remarkable capacity of resiliency, and as long as basic processes are not irretrievably upset, ecosystems will continue to recycle and distribute energy [9]. However, irreversible damages have been already caused due to inadequate management of the activity. The main negative impacts attributed to the activity are as follows.

**(1) Destruction of Natural Ecosystems, In Particular Mangrove Forests to Construct Aquaculture Farms [4, 10, 11]**

The mangrove forests are important ecosystems considered as the main source of organic matter to the coastal zone [12, 13]; they are also nursery areas for many aquatic species ecologically and/or economically important, as well as refuge or nesting areas for bird, reptiles, crustaceans, and other taxonomic groups [14]. Mangroves are additionally accumulation sites for sediments, contaminants, nitrogen, carbon and offer protection against coastal erosion [15]. According to environmentalists [16], mangroves support diverse local fisheries and also provide critical nursery habitat and marine productivity which support wider commercial fisheries. These forests also provide valuable ecosystem services that benefit coastal communities, including coastal land stabilization and storm protection.

The cover of mangrove forest has decreased worldwide from 19.8 million hectares in 1980 to less than 15 millions in 2000. The annual deforestation rate was 1.7% from 1980 to 1990 and 1.0% from 1990 to 2000 [17], and the problem continues up today. Some authors have documented that aquaculture has been responsible for the deforestation of millions hectares of mangrove forest in Thailand, Indonesia, Ecuador, Madagascar, and other countries [18, 19]. From 1975 to 1993, the construction of shrimp farms in Thailand diminished the mangrove cover from 312,700 to 168,683 ha [20]. Philippines has reconverted 205,523 ha of mangrove and wetlands into aquaculture farms, Indonesia 211,000 ha, Vietnam 102,000 ha, Bangladesh 65,000 ha, and Ecuador 21,600 ha [21].

**(2) Salinization/Acidification of Soils**

Aquaculture farms are sometimes abandoned by multiple problems (operative, economic, sanitary, and etc.), and the soil from those former farms remain hypersaline, acid and eroded [22]. Therefore, those soils cannot be used for agricultural purposes and are unusable for long periods. In addition, the application of lime and other chemicals used in aquaculture to treat the soil can also modify its physicochemical characteristics, which could aggravate the problem [23].

**(3) Pollution of Water for Human Consumption**

Although few studies have been conducted in relation with such topic, there are some signs indicating that inland aquaculture has been responsible for the deterioration of water bodies used for human consumption [21]. For instance, preliminary calculations revealed that an intensive aquaculture system farming three tons of freshwater fish can be compared, in respect to waste generation, to a community of around 240 inhabitants [24].

Although most of the aquaculture farms produce marine species, there is a growing sector of aquaculture farms producing freshwater species, which is a point of concern considering the above information.

**(4) Eutrophication and Nitrification of Effluent Receiving Ecosystems**

The eutrophication or organic enrichment of water column is mainly produced by nonconsumed feed (especially due to overfeeding), lixiviation of aquaculture feedstuffs [25, 26], decomposition of died organisms, and overfertilization [27–30]. It is well documented that from the total nitrogen supplemented to the cultured organisms, only 20 to 50% is retained as biomass by the farmed organisms, while the rest is incorporated into the water column or sediment [31, 32], and eventually discharged in the effluents toward the receiving ecosystems, causing diverse impacts such as phytoplankton blooms (sometimes of toxic microalgaes, such as red tides) [33], burring, and death of benthic organisms, as well as undesirable odors and the presence of pathogens in the discharge sites [34]. The impact may be more or less severe depending on some factors such as the intensification of the system (density of organisms), which is directly related to the amount of feed supplied [26, 35]. The feed conversion ratio (FCR) is a well indicator of the effectiveness of feeding and, consequently, of the retention of nitrogen and carbon as biomass of the farmed organisms. For instance, farms culturing the tiger shrimp *Penaeus monodon* usually report FCRs ranging from 1 to more than 2.5; such huge difference is later reflected in the amount of organic matter, nitrogen, and phosphorous discharged in the effluents, which may range from 500 to 1625 kg, 26 to 117 kg, and 13 to 38 kg, respectively, for each ton of shrimp harvested [28]. The estimated mean FCR worldwide for shrimp aquaculture is 1.8, which means that, for a world annual shrimp production around 5 million tons, 5.5 million tons of organic matter, 360,000 tons of nitrogen, and 125,000 tons of phosphorous are annually discharged to the environment. Unfortunately, these data considers only shrimp production, which represents around 8% of the total aquaculture production; if we assume that the FCRs are similar for the other farmed organisms and the diet formulations have some similitude [36], the total discharge of wastes may be multiplied by 12.5 from a very preliminary perspective. The nutrification is considered as the nutrient (N, P, C) enrichment of water column, mainly due to fertilization, mineralization of organic matter, resuspension of sediments, and excretion of organisms into the ponds. The greatest concern in this aspect is the increasing production of nitrogenous metabolites especially ammonia, which is highly toxic in its unionized form (NH_3_) for many aquatic organisms [37].

**(5) Ecological Impacts in Natural Ecosystems because of the Introduction of Exotic Species**

The negative impacts of the “biological contamination” for the introduction of exotic aquacultural species on the native populations have been well documented [18, 38, 39]. The main reported problems are the displacement of native species, competition for space and food, and pathogens spread. To cite an example, recent reports have revealed a parasite transmission of sea lice from captive to wild salmon [40]. The authors of such study have hypothesized that “if outbreaks continue, then local extinction is certain, and a 99% collapse in pink salmon abundance is expected in four salmon generations.”

**(6) Ecological Impacts Caused by Inadequate Medication Practices**

Farmers usually expose their cultured organisms to medication regimes, for different purposes such as avoiding disease outbreaks and improving growth performance. However, monitoring studies have detected low or high levels of a wide range of pharmaceuticals, including hormones, steroids, antibiotics, and parasiticides, in soils, surface waters, and groundwaters [41]. These chemicals have caused imbalances in the different ecosystems. In particular, the use of hormones in aquaculture and its environmental implications have been scarcely studied.

**(7) Changes on Landscape and Hydrological Patterns**

The agricultural and aquacultural activities have contributed to the degradation of ecosystems including important modification on landscape [10, 18, 22, 42]. The construction of shrimp farms in the river beds has modified the hydrological patterns in many regions of the world with the consequent impacts on the regional ecosystems and the local weather.

**(8) Trapping and Killing of Eggs, Larvae, Juveniles, and Adults of Diverse Organisms**

It has been estimated that, for each million of shrimp postlarvae farmed, four to seven millions of other organisms are killed by trapping in the nets of farms inlet [18, 43].

**(9) Negative Effect on Fisheries**

Although aquaculture has been proclaimed as a solution to avoid overfishing, it has contributed in more or less proportion to the fisheries collapse. Fishermen who work in places near to aquaculture farms argue that the contamination produced by farms has decreased the population of aquatic organisms and in consequence their volume captures. Additionally, another problem of similar magnitude is the extremely high aquaculture's dependence of fishmeal and fish oil, which could be another nonsustainable practice in aquaculture. The proportion of fishmeal supplies used for fish production have increased from 10% in 1988 to more than 30% in the last years, which classifies aquaculture as a potential promoter of the collapse of fisheries stocks worldwide [24].

**(10) Some Other Accusations**

Some other accusations for aquaculture include the production of fish and shellfish with high concentrations of toxins and/or heavy metals; genetic pollution and infestation of nondesirable phytoplankton and/or zooplankton species [44–47].

**(11) In Its Role as Food Producer, Aquaculture Is Far from Complying an Adequate Distribution of Food**

Overlaying net exports, governance, and undernourishment suggest that “seafood's contribution as a source of protein and livelihood is precarious” [6]. Moreover, it has been revealed that some countries with undernourishment and weak governance usually play a role as baler of seafood from countries well nourished and with strong economic capacity [6].

For these above reasons remains a generalized perception that the sustainability of aquaculture is at present being threatened or, in some cases, far from being reached.

## 3. What to Do for a Sustainable Aquaculture?

Many strategies have been suggested, evaluated, and/or proven in order to advance in the sustainability of aquaculture. Basically, all of them respond to the criticisms and are possible solutions to the problems attributed to the activity. The main aspects that have to be performed to advance toward such goal are the correct selection of the farming sites and species; the implementation of the most adequate culture system; use of the best feed and feeding practices; the use of bioremediation systems; decreasing the dependence of fishmeal and fish oil; adequate management of the effluents; achieving certification of compliance with sustainability; improving research and legislation related to evaluation and solutions for aquaculture impacts.

(1) In the context of the site selection it is necessary to consider the following.

- The vocation of the selected site. It would be absurd to select a site for aquaculture purposes if it is excellent for agriculture or livestock. Unfortunately, this is the case in many regions of the world, where agricultural lands have been reconverted to aquaculture farms. The vocation of a selected site is determined for many aspects (which can change from region to region) such as physical and chemical soil characteristics; water availability, soil fertility, topography, wild vegetal and animal communities, proximity to cities, towns, tourism zones, and so forth; priorities of the region or country (food, fuels, tourism budget, aquaculture budget, and etc.).
- The carrying capacity of the water bodies from the sites considered to supply the farms or used as effluent discharge places. It is very important to evaluate how much water can be taken from a particular water body or how much effluents it can receive without important alterations on its ecological equilibrium [48]. The use of advanced technologies such as remote sensing could be an excellent auxiliary in this field [11].

(2) For the selection of species it is crucial to consider the following.

- It is always better to select native instead of exotic species. The introduction of exotic species causes many and diverse problems as mentioned in the previous section. Additionally, the obtaining and maintenance of broodstock of exotic species could be difficult and expensive.
- It is necessary to have the most possible knowledge about the biology and ecology of the organism that is pretended to be farmed (life cycle, feeding habits and nutritional requirements, tolerance to environmental parameters, and etc.).
- It is important to select organisms with a good market and price when farmed for commercial purposes.

(3) Regarding implementation of the best culture system, the main aspects to consider include the following.

- The type and size of farming structure [49]. Depending on the species, intensity, land and water availability, and economic investment, it is possible to use different types of farming structures for the culture of the same species or group. Some of them are more adequate and sustainable. For the case of shrimp farming, for instance, it has been suggested that floating or submerged cages could have a lower impact on the environment than earthen ponds. The same suggestion is applicable for culture of fishes or mollusks. Regarding size of production units, small ponds or farming structure is easier to manage in aspects such as feeding, monitoring, cleaning, pond bottom management, and harvesting. Such considerations usually lead to lower environmental impacts.
- Intensity. The stocking density and the consequent biomass harvested are absolutely related to the sustainability of aquaculture. The increase of the intensity implies an increase in the supplemental feed and in consequence, in the organic matter, nitrogen, and phosphorous in the effluents. Additionally, intensive or super intensive systems require the use of diverse chemicals (antibiotics, algaecides, parasiticides, and etc.), which also contribute to increasing the pollution [50]. The most adequate intensity depends on the land and water availability, as well as the carrying capacity of the water body or terrestrial ecosystems which will receive the effluents. However, recalculating and zero water exchange systems can eliminate the environmental impact while maintaining extremely high densities of aquatic organisms. Promising results have been achieved in the culture of fish and crustaceans using biofloc systems with zero water exchange [51].
- An adequate design of the water inlet and outlet systems, considering the water quality, weather conditions, marine currents and tide patterns (for sea water), and hydrological patterns (for continental waters) [52]. The modifications of oceanic currents patterns may have implications on the sediment transport and consequently on the beaches conformation.
- The possibility of farming simultaneously two or more species (polycultures or integrated multitrophic aquaculture (IMTA)). This strategy has proven to be one of the most effective ways to recuperate the carbon, nitrogen, and phosphorous supplied to the system as biomass of the farmed organisms and to diminish the environmental impacts caused by the effluents [53–55]. Polyculture is commonly referred to organisms of the same environment (marine, brackish wáter, or continental waters) and trophic level, while IMTA is mostly referred to organisms from different trophic levels and inclusively different environments. The implementation of such alternative systems improves the nutrient cycling within the culture units. In short, while in a traditional aquaculture system, 25 to 35% of the nitrogen supplied is recuperated as biomass of the farmed organisms, in a polyculture or IMTA, the recuperation could be increased by more than 50%. A pilot project made aware and informed a group of participants about the benefits of IMTA; the authors revealed that 50% of the participants were willing to pay an extra 10% of products labeled as of “IMTA products.” Moreover, the authors were optimistic regarding the social impacts caused by the implementation of IMTA as a sustainable practice [5].

(4) Since supplemental feed is considered the main source of contamination of aquaculture systems and effluent receiving ecosystems [56], the improvement of these feed, as well as the feeding, strategies could be considered as an important part of the solution for a sustainable aquaculture [28, 57]. The main aspects in which the feedstuffs must be improved include the following.

- Better and more precise formulations for the particular species to be farmed, which consider the best concentration and quality of the nutrients. A common practice of world aquaculture is the use of diets with protein contents higher than those required, thus affecting not only the price of the feed but also increasing the pollution potential, considering that protein catabolism produces ammonium nitrogen as the main metabolite. Regarding nutrient quality, it is important to use ingredients with high digestibility; the low digestibility of ingredients (protein, lipid, carbohydrate) is partially the responsible for a low retention of those nutrients in the farmed organisms and their increase in the water column and sediment, augmenting the polluting potential [58].
- Higher hydrostability. One of the most important causes of nutrient losses of aquafeeds is the low hydrostability, which provoke fast disintegration and lixiviation, decreasing the nutrient incorporation efficiency by the farmed organisms and increasing the concentration in the water column. Fishes are faster swimmers and can consume a formulated feed within minutes, but crustaceans are usually less active and can consume the formulated feed within minutes or even hours. The hydrostability of feedstuffs can be improved by incorporation of effective binders and/or for the use of special fabrication processes [59].
- Better attractability and palatability. It is necessary to produce feeds which can be consumed as soon as possible to avoid nutrient losses. This is possible with the incorporation of effective attractants and improving the palatability with ingredients such as fish oils and others. Many of these ingredients have been sufficiently proven [60].

(5) Regarding to the feeding strategies some important advances have been achieved but there are yet much more to advance in aspects such as forms to supply the feed, adjustment of the ration, and frequency of feeding.

- The use of feeding trays and the increase of feeding frequency have been demonstrated to diminish the pollution potential of the effluents in shrimp farms [37]; however these strategies are suitable only for high-intensity systems (intensive or superintensive), but not economically feasible for extensive, semiextensive of semiintensive systems.
- The promotion, management, and rational utilization of natural feed, including microorganisms (biofilm, biofloc), are considered as a promising strategy for the culture of shrimp, fishes, and mollusks. Some authors [61–63] have successfully enhanced the production of zooplankton and benthos in shrimp ponds and demonstrated their great contribution not only in the production response, but also in the nutritional, sanitary, and immune condition of the farmed organisms. Additionally, the use and contribution of microorganisms associated to biofilms and bioflocs for the nutrition of farmed organisms have been also documented [24, 64–66]. Such practice may also decrease the dependence of fishmeal and fish oil; however other strategies such as the use of plant ingredients and the use of bioflocflour have been tested and proposed to substitute at different rates the fishmeal in formulated feeds [51].
- The practice of subfeeding or intermittent-feeding regimes is a strategy aimed to achieve average growth performances in aquatic organisms, but supplying significantly lower amounts of formulated feed. Such alternative takes advantage of the compensatory growth process of shrimp and crustaceans [67].

(6) The adequate management of effluents is indubitably one of the central aspects to consider for a sustainable aquaculture. Diverse strategies have been proven or suggested to minimize the environmental impacts of effluents. The most promising are settling lagoons [34], treatments with septic tanks [68], the implementation of systems with low or zero water exchange [69], the utilization of recirculation systems [70, 71], the use of mangrove forests as sinks for nutrients, organic matter, and contaminants [72], the polyculture or integrated multitrophic aquaculture systems [55, 73], and the bioremediation [54, 74].

- It is considered as bioremediation the use of individual or combined organisms (including animal, vegetal, and bacteria) to minimize the contaminating charge of effluents from any activity (including aquaculture). This practice takes advantage of the natural or modified abilities of those organisms to reduce and/or transform waste products [75].
- There are different ways to conduct bioremediation: in situ, ex situ, biostimulation, bioaugmentation, and others. Many successfully examples of bioremediation practices can be mentioned: the use of plants (phytoremediation), macroalgae, microalgae, filter feeders, biofilters (polymer spheres with immobilized microorganisms), biofilms, and bioflocs [76, 77]. There are also combined systems which use two or more of these practices. Many studies have been conducted to use individual or combined organisms for bioremediation [78–80]. However, the ideal strategy would be the decreasing or complete halting of effluent discharge and using zero water exchange systems.

(7) Achieve certification of compliance with sustainability.

A combination of analyses has been suggested to evaluate the sustainability of commercial aquaculture farms [81]. For example, the authors of such contribution suggest the calculation of mass balances and undesirable outputs of shrimp farms; calculation of the input distance function approach which provides a complete characterization of the structure of multiinput, multioutput efficient production technology and provides a measure of the distance from each producer to that efficient-sustainable technology; finally a productivity measurement with and without undesirable outputs. However, the analysis of the socioeconomic impacts caused by farms ought to be included in the list.

Additionally, certification processes can be followed to assure the sustainability of aquaculture or to compare the standards established by the different agencies and check if the practices of any farm cope with those standards. The certification of aquaculture is performed by the International Standards Organization (ISO), the WTO Technical Barriers to Trade (TBT), the FAO Guidelines for the Ecolabelling of Fish and Fishery Products from Marine Capture Fisheries, and the Network of Aquaculture Centres in Asia-Pacific (NACA) and others [82].

According to the FAO criterion [82], certification “is a procedure through which written or equivalent assurance states that a product, process, or service conforms to specified requirements. Within the aquaculture sector certification can be applied to a process followed by a production unit (pond, cage, farm, processing plant), a specific product or commodity or to the inputs being applied to the system before or during production.”

 (8) Finally, there is an unavoidable need to improve research and legislation regarding evaluation and solutions for aquaculture impacts.

- One of the reasons of the severe environmental impacts of aquaculture is that scientific research in some developing countries is firstly focused on increasing biomass production (improvement of formulated feeds, production systems, genetically improved organisms, etc.) and later on the environmental impacts; however, it is desirable to evaluate the potential impacts of any farm that is pretended to be installed, rather than monitoring the pollution that is already being caused by any farm constructed without considering its environmental impact.
- In addition, there is a great heterogeneity regarding policies and legislation of aquaculture impacts among different countries; while some developed countries have complete and concrete legislation for aquaculture in order to avoid environmental impacts, others have weak policies that do not protect their environment from aquaculture wastes; under such scenario ecological imbalances and disasters have been caused, with some of them being irreversible. Herein, Smith et al. asserted that “some developing countries often lack the institutions necessary to prevent deleterious ecosystem impacts of seafood production and to sustain trade benefits” [6]; they also argued that “the developed countries have a history of these problems as well, but with less-obvious consequences.” In the same report, the authors revealed that with base in the World Bank indicators, more than 60% of the countries had inefficient governance regarding the regulation of aquaculture and fisheries activities; a possible cause of such result is related to corruption and regulatory quality. Thus, it is absolutely essential for the future of aquaculture that the governments and the producers be attuned with each other to reach agreements that resolve the problems of this activity.
- Finally, contrasting actions have been observed by governmental instances, while some instances try to protect the environment and achieve a sustainable aquaculture, others have directly or indirectly supported the unsustainable aquacultural practices; for instance, according to a recent report [16] “the conversion of mangroves to aquaculture ponds has been fuelled by governmental support, private sector investment and external assistance from multilateral development agencies such as the World Bank and Asian Development Bank” [83, 84].

In conclusion, aquaculture is a possible panacea, but at present is also responsible for diverse problems related with the environmental sanity; however the new strategies proposed during the last decade have proven that it is possible to reach a sustainable aquaculture, but such strategies should be supported and proclaimed by the different federal environmental agencies from all countries. Only under such scenario, aquaculture will be a sustainable practice. The implementation of the different alternatives stated above would depend on particular circumstances of any farm. Fortunately, there are reports of some aquaculture farms along the world on sustainable practices.
